# An evidence-gated strategy for translating targeted oncologic PET radiopharmaceuticals from molecular design to clinical use

**DOI:** 10.3389/fnume.2026.1976156

**Published:** 2026-10-27

**Authors:** Qiheng Xiong, Wen He, Deshun Fu, Yu Su

**Affiliations:** Department of Nuclear Medicine, Jiangxi Cancer Hospital, Nanchang, Jiangxi, China

**Keywords:** fibroblast activation protein, molecular design, PET radiopharmaceuticals, pharmacokinetics, PSMA, radiochemistry, somatostatin receptor, theranostics

## Abstract

Targeted oncologic positron emission tomography (PET) radiopharmaceutical development is often presented as a linear progression from target discovery to clinical imaging. Translation instead depends on interdependent biological, chemical, pharmacokinetic, manufacturing, and regulatory decisions. This Review examines those decisions as an integrated design problem. We consider how target accessibility and heterogeneity, vector format, linker and chelator chemistry, radionuclide properties, and whole-body pharmacokinetics jointly determine image contrast and normal-organ exposure. We then assess how radiosynthesis, analytical validation, molar activity, automation, and batch release determine whether a promising construct can become a reproducible clinical product. Prostate-specific membrane antigen (PSMA) and somatostatin receptor platforms show how molecular imaging can support patient selection for radionuclide therapy when the diagnostic agent, therapeutic construct, trial population, and regulatory indication are aligned. By contrast, fibroblast activation protein (FAP)-targeted and other emerging platforms show why biodistribution or early human feasibility cannot establish clinical benefit. Across these examples, affinity alone is an insufficient design objective. The relevant endpoint is a clinically usable exposure profile that preserves target uptake while limiting background retention and dose to critical organs. Major unresolved issues include inconsistent head-to-head evaluation, incomplete dosimetry, product-specific manufacturing constraints, heterogeneous evidence standards, and region-dependent regulatory indications. We propose an evidence-gated framework that separates target suitability, molecular exposure, manufacturability, human performance, and implementation. This framework distinguishes established practice from early investigation and identifies studies most likely to reduce translational uncertainty.

## 1 Introduction

PET can measure the spatial and temporal distribution of radiolabelled compounds at tracer concentrations, providing information that is not available from anatomical imaging alone. Its clinical value, however, does not arise from the imaging device in isolation. It depends on whether a radiopharmaceutical can interrogate a biologically relevant process with adequate target-to-background contrast, acceptable radiation exposure, and a synthesis and quality-control procedure that can be reproduced within the short time constraints imposed by radioactive decay. Recent reviews of cancer PET have accordingly moved beyond cataloguing tracers and instead treat probe design, quantitative imaging, instrumentation, and clinical interpretation as interdependent components of molecular imaging (1).

The expansion of radiotheranostics makes this interdependence more consequential. A diagnostic scan may be used to establish target expression and disease distribution before a chemically related or biologically matched therapeutic radiopharmaceutical is administered. Randomized trials of [^177^Lu]Lu-PSMA-617 and [^177^Lu]Lu-DOTA-TATE have shown that this strategy can improve clinically relevant outcomes in selected patients with metastatic castration-resistant prostate cancer and neuroendocrine tumours, respectively (2–5). These successes do not establish that any imaging ligand can be converted into an effective therapeutic agent. They instead show that target biology, ligand pharmacokinetics, radionuclide emissions, administered mass, absorbed dose, prior therapy, and prespecified PET acquisition and interpretation criteria used to determine treatment eligibility must be evaluated as one clinical system.

This distinction is easily lost when “next-generation” radiopharmaceuticals are described primarily by target affinity or image appearance. Molecular changes that improve binding can also alter plasma protein binding, metabolism, clearance, or retention in kidneys, liver, salivary glands, marrow, and other dose-limiting tissues. For therapeutic constructs, the classical tracer assumption is further strained because the objective is not merely to observe a biological process but to deliver cytotoxic radiation while preserving an adequate therapeutic window. Contemporary design work therefore focuses increasingly on controlling distribution and metabolism through vector format, charge, hydrophilicity, chelation, bioconjugation, and cleavable or otherwise metabolically responsive linkers (6). The relevant comparison is not “high versus low affinity” in isolation, but whether an integrated molecular construct improves tumour exposure relative to normal-organ exposure on a time scale compatible with the chosen radionuclide.

Manufacturability imposes an additional translational filter, distinct from biological performance. A compound with favourable preclinical uptake may still fail to translate if its precursor is difficult to obtain, labelling is sensitive to small changes in reagent quality, purification cannot be automated, molar activity varies between batches, or analytical methods cannot establish identity, purity, and stability within a clinically useful release window. EANM guidance on analytical-method validation, molar activity, and ^68^Ga production underscores that radiopharmaceutical quality is a measured property of a defined process and product, not an assumption derived from a high radiochemical yield (7–9). Product-specific pharmacopoeial and regulatory requirements must therefore be integrated during development, not added after biological optimization.

The clinical evidence is also uneven. PSMA- and somatostatin receptor-targeted platforms now include randomized therapeutic trials and region-specific regulatory authorizations, whereas many FAP/FAPI and other emerging agents remain supported mainly by biodistribution, dosimetry, diagnostic-performance, or small early-phase treatment studies. These evidence types answer different questions and should not be placed on a single undifferentiated continuum of ‘promise’. In the United States, the indication for [^177^Lu]Lu-PSMA-617 was expanded in 2025 following PSMAfore (4, 10). At the review cut-off, the European Medicines Agency product description remained framed differently (11). Regulatory statements must therefore identify the jurisdiction and verification date.

This Review develops an evidence-gated account of targeted oncologic PET radiopharmaceutical translation from molecular architecture to clinical theranostics. It asks which target and molecular-design features are necessary but insufficient for translation, how radiochemistry and quality systems constrain clinical use, and what separates established PSMA and somatostatin receptor platforms from earlier-stage FAP/FAPI and other targets. The selected platforms are representative tests of different translational constraints, not an exhaustive account of neurological, cardiovascular, metabolic, perfusion, or infectious-disease PET. The aim is to connect chemical design choices to measurable pharmacokinetic, manufacturing, dosimetric, and clinical consequences while separating established findings, author synthesis, and unresolved hypotheses. Figure 1 introduces the six author-derived decision gates, Figure 2 shows linked molecular modules and verified representative parent-ligand structures, and Table 1 summarizes the representative platforms and the highest level of evidence identified for each question.

**Figure 1:**
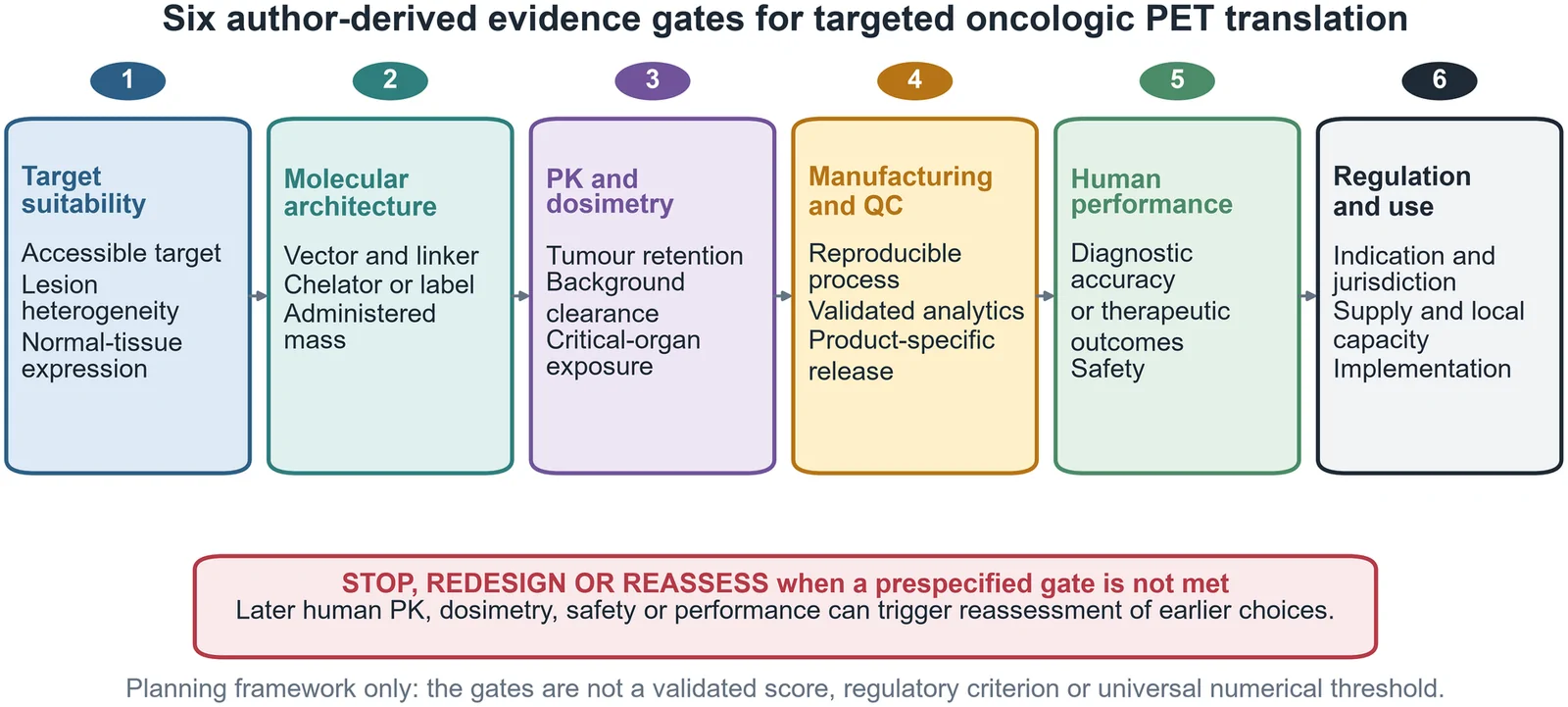
Six author-derived evidence gates for targeted oncologic PET translation. Six author-derived decision gates separate target suitability, molecular architecture, pharmacokinetics and dosimetry, manufacturing and quality control, human performance, and regulation and implementation. Forward arrows indicate conditional progression, not a validated score or deterministic sequence. Human pharmacokinetic, dosimetric, safety or performance data can trigger reassessment of earlier choices. The framework is a planning synthesis rather than a regulatory acceptance criterion or universal numerical threshold; its evidence basis is described in Section 2 and in the cited design, manufacturing and clinical sources.

**Figure 2:**
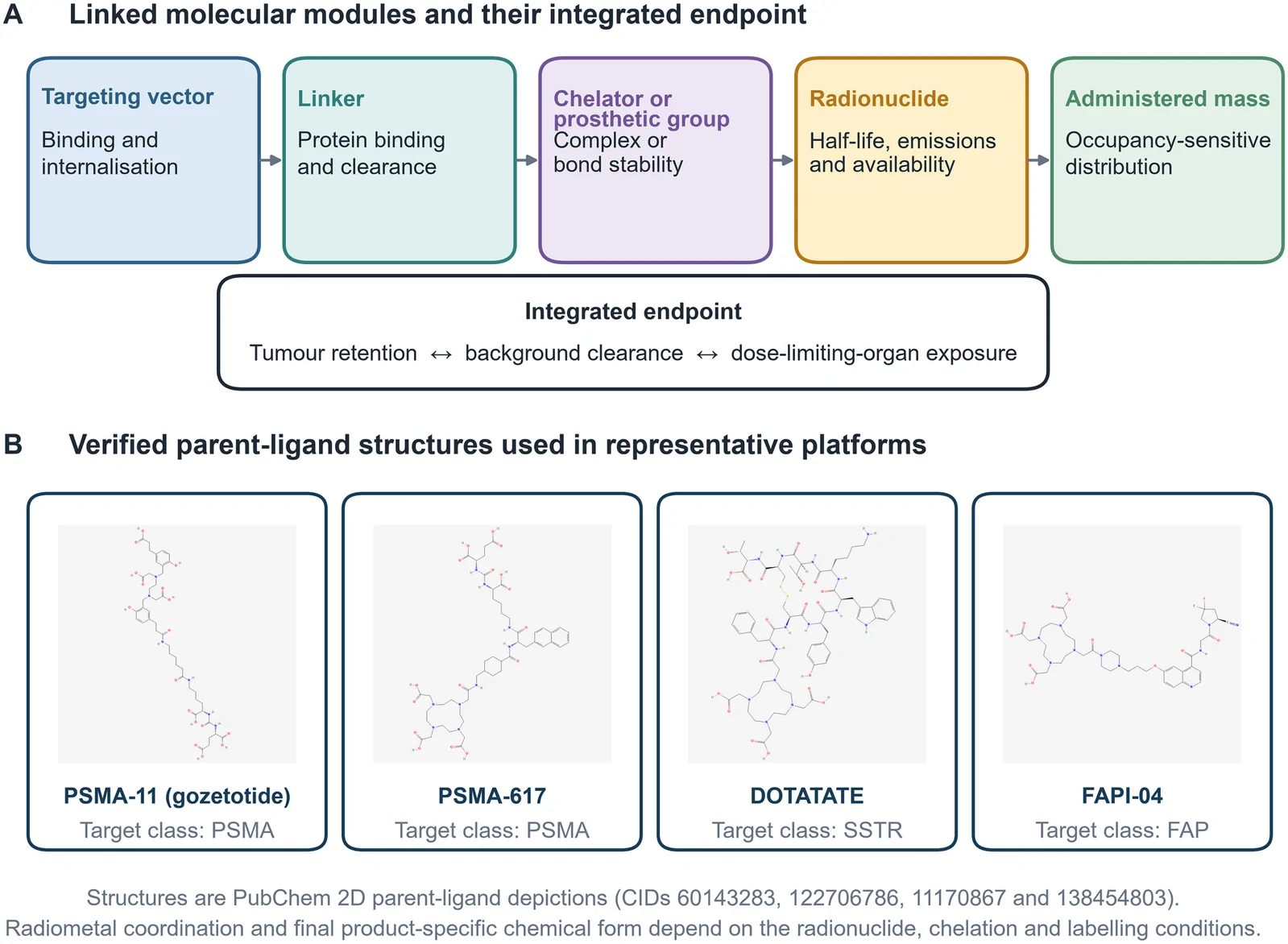
Linked molecular modules and verified representative parent-ligand structures. **(A)** Linked molecular modules—targeting vector, linker, chelator or prosthetic group, radionuclide, and administered mass—jointly determine tumour retention, background clearance and dose-limiting-organ exposure. **(B)** Verified PubChem two-dimensional parent-ligand depictions for PSMA-11 (gozetotide; CID 60143283), PSMA-617 (CID 122706786), DOTATATE (CID 11170867) and FAPI-04 (CID 138454803). These are parent-ligand depictions; radiometal coordination and the final product-specific chemical form depend on the radionuclide, chelation and labelling conditions. Structure source: PubChem (67).

**Table 1:** Representative PET-targeted platforms and the maturity of their theranostic evidence.

Target/platform	Representative PET agent	Representative therapeutic agent	Vector/label class	Highest verified human evidence	Regulatory status at evidence cut-off	Clinically relevant limitation	Key sources
Prostate-specific membrane antigen (PSMA)	[^68^Ga]Ga-gozetotide (PSMA-11); [^18^F]piflufolastat	lutetium Lu 177 vipivotide tetraxetan ([^177^Lu]Lu-PSMA-617; Pluvicto)	Urea-based small-molecule ligand; radiometal chelation or covalent ^18^F labelling	Randomized phase 3 treatment evidence in post-taxane mCRPC (VISION) and taxane-naive mCRPC after an androgen-receptor pathway inhibitor (PSMAfore)	US: PET agents and [^177^Lu]Lu-PSMA-617 authorized; the therapeutic indication was expanded on 28 March 2025 to selected patients for whom taxane delay is appropriate. EU: Locametz and Pluvicto centrally authorized; Pluvicto remained framed for prior AR-pathway inhibition and taxane exposure on the EMA page checked.	PSMA-negative or heterogeneous disease, agent-specific physiologic/off-target uptake, and trial-specific PET eligibility rules prevent a single universal positivity threshold.	Sartor et al. (2); Morris et al. (4); Fendler et al. (47); Kratochwil et al. (48); FDA (10); EMA (11, 56)
Somatostatin receptor (SSTR)	[^68^Ga]Ga-DOTATATE; [^64^Cu]Cu-DOTATATE	lutetium Lu 177 dotatate ([^177^Lu]Lu-DOTA-TATE; Lutathera)	Cyclic peptide with DOTA chelation	Randomized phase 3 treatment evidence in progressive midgut NETs (NETTER-1) and first-line advanced grade 2–3 well-differentiated GEP-NETs (NETTER-2)	US: NETSPOT, Detectnet and Lutathera authorized; Lutathera was extended in 2024 to patients aged ≥12 years with SSTR-positive GEP-NETs. EU: Lutathera centrally authorized for adults with unresectable/metastatic, progressive, well-differentiated G1-G2 SSTR-positive GEP-NETs.	SSTR-negative clones, grade/differentiation, renal and marrow exposure, and differences between diagnostic uptake and deliverable therapeutic dose constrain transfer across settings.	Strosberg et al. (3, 34); Singh et al. (5); FDA (35, 57, 58); EMA (59)
Fibroblast activation protein (FAP)	[^68^Ga]Ga-FAPI-04	[^177^Lu]Lu-FAP-2286 and [^177^Lu]Lu-EB-FAPI (investigational examples)	Quinoline-based small molecules or cyclic peptide; DOTA chelation; optional albumin binder	Multicancer human imaging/biodistribution and small uncontrolled first-in-human or dose-escalation treatment studies; no randomized comparative therapeutic trial was identified in the targeted search	No centralized FDA or EMA authorization for a FAP-targeted radiopharmaceutical was identified in the official pages searched.	Uptake varies between and within tumour types; wound healing, fibrosis and inflammation can be FAP-avid; early treatment series cannot establish comparative efficacy.	Giesel et al. (17); Kratochwil et al. (36); Baum et al. (38); Fu et al. (39)
Human epidermal growth factor receptor 2 (HER2)	[^89^Zr]Zr-trastuzumab; [^68^Ga]Ga-ABY-025	No FDA/EMA-authorized HER2-targeted radiopharmaceutical therapy pair identified in the bounded search	Full antibody with desferrioxamine chelation; engineered Affibody with chelation	Small prospective biodistribution/dosimetry and lesion-detection studies	No centralized FDA or EMA authorization for either representative HER2 PET agent was identified in the official pages searched.	Antibody imaging requires delayed acquisition and increases whole-body exposure; engineered proteins clear faster but can have high renal uptake, and administered peptide mass can alter distribution.	Dijkers et al. (42); Ulaner et al. (43); Sandstrom et al. (44)
C-X-C chemokine receptor 4 (CXCR4)	[^68^Ga]Ga-pentixafor	[^177^Lu]Lu- or [^90^Y]Y-pentixather (investigational examples)	Cyclic pentapeptide; radiometal chelation	Human staging studies and small first-in-human endoradiotherapy experience	No centralized FDA or EMA authorization for the representative CXCR4 agents was identified in the official pages searched.	Heterogeneous and temporally variable target expression, physiologic haematopoietic expression, and marrow exposure complicate selection and therapy.	Shekhawat et al. (45); Herrmann et al. (46)

In this Review, an ‘established’ platform has a reproducible selection method, randomized therapeutic evidence, and at least one region-specific authorization for a defined population. ‘Highest verified human evidence’ describes the strongest evidence identified for the platform and does not imply that every listed agent has that evidence. Regulatory statements are date- and jurisdiction-specific; current prescribing information governs clinical use. US and EU product pages were checked; the China NMPA source was used only to define the national framework, not to make product-level status claims (12). mCRPC, metastatic castration-resistant prostate cancer; AR, androgen receptor; NET, neuroendocrine tumour; GEP-NET, gastroenteropancreatic neuroendocrine tumour.

## 2 Scope, search strategy and evidence appraisal

This article is a narrative Review; it is neither a systematic review nor a meta-analysis. Targeted searches covered 1 January 2000 to 9 August 2026 in PubMed and on publisher websites. Search concepts combined radiopharmaceutical or radiotracer, PET and oncology with target- or platform-specific terms and with design, linker, chelator, pharmacokinetics, dosimetry, manufacturing, quality control, regulation or clinical trial. Earlier seminal sources were retained when identified by backward citation checking. Eligible sources were peer-reviewed original studies, clinical trials, authoritative procedure or radiopharmacy guidelines, and official regulator or technical pages in English for which the product, population or setting could be identified. Regulatory claims were checked against US Food and Drug Administration, European Medicines Agency and, at framework level, China National Medical Products Administration sources (10–12).

Clinical and regulatory statements were prioritized as follows: current regulatory labels and official assessment pages; randomized trials; prospective multicentre studies; prospective single-centre studies; retrospective studies; first-in-human or dosimetry studies; and preclinical experiments. This hierarchy applies to clinical or regulatory claims, not to mechanistic chemistry questions for which a paired preclinical comparison can be the most direct design. Reviews were used to frame concepts and identify primary literature, but treatment-effect claims were traced to the relevant trial whenever possible. Only published or formally accepted material was eligible. Conference abstracts, company promotional material, unpublished data, unverifiable claims, and non-oncologic applications outside the stated scope were excluded.

PSMA and somatostatin receptor platforms were selected as mature comparators, FAP/FAPI as an emerging stromal platform, and HER2 and CXCR4 as focused examples of vector-kinetic and marrow-risk constraints. This purposeful selection introduces platform-selection and publication bias and is not an exhaustive inventory of targets, radiopharmaceuticals or jurisdictions. Because the Review spans chemistry, production and clinical application, one hierarchy was not applied to every question. The six gates—target suitability, molecular architecture, pharmacokinetics and dosimetry, manufacturing and quality control, human performance, and regulation and implementation—were derived by mapping recurring translational decisions across the included source types. Each gate asks a different decision question and is linked to a minimum evidence type, an endpoint and a stop or redesign trigger. The framework is an author-derived decision aid, not a validated score, regulatory acceptance criterion or universal numerical threshold.

## 3 Molecular design as an exposure-control problem

### 3.1 Target suitability extends beyond expression level

A candidate target is useful only if it is accessible to the selected vector and produces a clinically interpretable difference between disease and relevant background tissues. High expression in an excised tumour sample is therefore insufficient. The distribution of the target across lesions, its presence in normal organs, membrane accessibility, internalization and recycling, and changes induced by prior therapy can all alter the relationship between measured uptake and disease biology. These factors matter differently for diagnostic and therapeutic applications. Moderate normal-organ uptake may still permit diagnostic lesion detection, whereas prolonged retention in the same organ can constrain the administered activity of a therapeutic agent. Established radiotheranostic platforms illustrate that patient selection is a biological and operational definition: the imaging agent, acquisition and interpretation rule, disease setting, and therapeutic construct must be considered together (13).

Target heterogeneity creates two distinct problems. Between-patient heterogeneity determines how many patients are eligible for a target-directed strategy, while within-patient heterogeneity determines whether an imaging-positive subset of disease adequately represents the total tumour burden. A single patient-level label such as “target positive” can conceal lesion-level discordance. For this reason, a development programme should specify whether the intended use is lesion detection, whole-patient selection, response assessment, or treatment delivery. Each use requires a different reference standard and a different tolerance for false-negative disease.

### 3.2 Vector format sets the biological time scale

Small molecules, peptides, engineered proteins, and intact antibodies cannot be compared only by affinity. Their sizes and molecular architectures lead to different rates of extravasation, target binding, blood clearance, metabolism, and excretion. Rapidly clearing small molecules and peptides often permit same-day imaging, whereas antibodies usually require delayed acquisition to allow blood-pool activity to decline (1, 6). These are class tendencies, not fixed rules; charge, glycosylation, protein binding, target-mediated disposition, and the position and number of labels can materially change the behaviour of an individual construct.

The radionuclide should be selected according to the pharmacokinetic time scale of the complete radiopharmaceutical rather than the unconjugated vector alone. Physical half-life is one constraint, but positron-emission characteristics, radiation burden, availability, production scale, labelling conditions, *in vivo* stability, and the intended imaging schedule also matter. Nomenclature should distinguish the radionuclide, radiolabelled compound, precursor, and measured radiochemical quantities because imprecise terms obscure which entity was evaluated (14). Human comparisons of ^68^Ga- and ^64^Cu-labelled somatostatin receptor ligands illustrate that radionuclide and construct choices change quantification and lesion-detection characteristics rather than acting as interchangeable labels (15, 16). For a radiometal, chelator compatibility and kinetic inertness are part of radionuclide selection; for a radiohalogen, the stability and metabolic fate of the labelled bond or prosthetic group are equally important. Figure 3 provides an orientation map across physical and biological time scales, and Table 2 summarizes the corresponding nuclear and operational properties.

**Figure 3:**
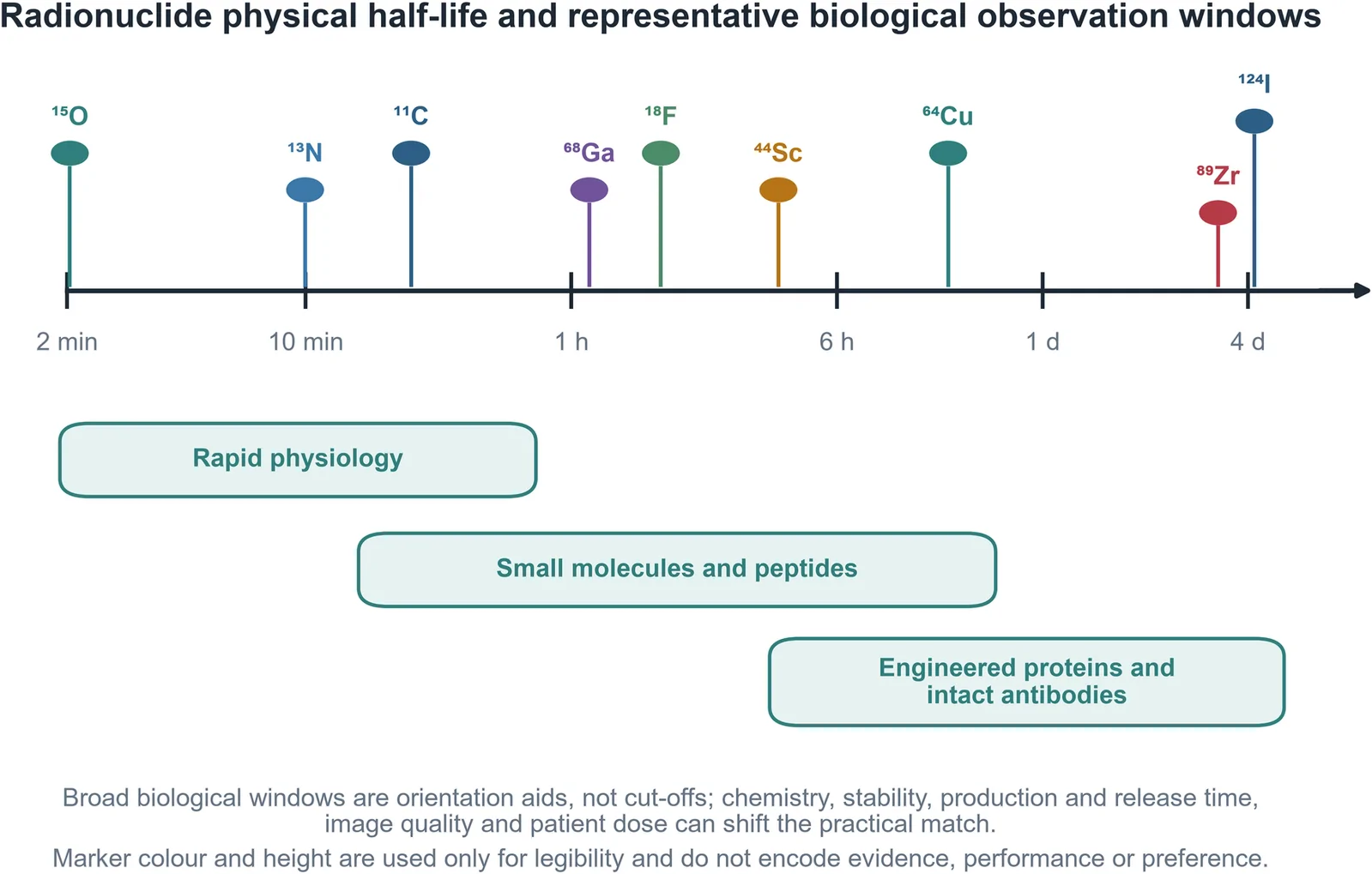
Radionuclide physical half-life and representative biological observation windows. Logarithmic time axis positioning selected PET radionuclides from ^15^O to ^124^I against approximate biological observation windows for rapid physiology, small molecules and peptides, and engineered proteins and intact antibodies. The bands are orientation aids rather than fixed categories. Marker colour and height are used only for label legibility and do not encode evidence, performance or preference. Numerical half-lives are taken from IAEA sources (60–62); production and operational constraints are summarized in Table 2.

**Table 2:** Nuclear and operational properties of selected PET radionuclides.

Nuclide	Physical half-life	*β*+ emission data total probability (%); highest endpoint (keV)	Production route	Labelling mode	Biological time-scale	Representative agent/use	Operational constraint
^11^C	20.364 min	99.7669%; 960.3 keV	^14^N(p,*α*)^11^C in a gas target	Covalent incorporation through [^11^C]CO2, [^11^C]CH4 or downstream synthons	Small molecules with rapid synthesis and imaging	[^11^C]methionine or [^11^C]choline	Requires an on-site cyclotron and rapid synthesis, release and imaging
^13^N	9.9584 min	99.803%; 1198.4 keV	^16^O(p,α)^13^N, commonly using water	Direct formulation or rapid conversion of target product	Very rapid perfusion/metabolic measurements	[^13^N]ammonia	On-site production and tightly synchronized quality control are essential
^15^O	122.24 s	99.9003%; 1731.9 keV	^14^N(d,n)^15^O or ^15^N(p,n)^15^O	[^15^O]water, oxygen or carbon-oxide gases	Near-real-time physiology and flow	[^15^O]water	The approximately 2-min half-life demands an integrated cyclotron-synthesis-scanner workflow
^18^F	109.77 min	96.73%; 633.4 keV	^18^O(p,n)^18^F using enriched [^18^O]water for [^18^F]fluoride	Nucleophilic/electrophilic covalent labelling; [^18^F]AlF chelation in selected systems	Small molecules and selected peptides; regional distribution is feasible	[^18^F]FDG; [^18^F]piflufolastat	Multistep radiosynthesis and impurity control must fit the available decay window
^44^Sc	4.0420 h	94.278%; 1473.6 keV	^44^Ca(p,n)^44^Sc; ^44^Ti/^44^Sc generator as a less widely available alternative	Chelation, commonly with DOTA-type ligands	Peptides and other vectors needing several-hour imaging	Clinical-development studies with ^44^Sc-labelled DOTA peptides	Co-emitted high-energy radiation, ^44^mSc control, target cost/recovery and limited routine supply
^64^Cu	12.7006 h	17.49%; 652.61 keV; mixed decay (beta-minus and electron-capture/positron branches)	^64^Ni(p,n)^64^Cu on enriched nickel	Chelation; kinetic stability of the Cu complex is product dependent	Peptides, small proteins and delayed imaging	[^64^Cu]Cu-DOTATATE	Enriched-target recovery, lower positron branching and *in-vivo* complex stability require explicit control
^68^Ga	67.71 min	88.9103%; 1890keV	^68^Ge/^68^Ga generator or ^68^Zn(p,n)^68^Ga cyclotron production	Chelation with DOTA, HBED-CC, NOTA or related systems	Small molecules and peptides with rapid targeting	[^68^Ga]Ga-DOTATATE; [^68^Ga]Ga-gozetotide	Generator throughput/breakthrough or cyclotron metal/radionuclidic impurities are route specific; the short half-life narrows batch-release time
^89^Zr	78.41 h	22.74%; 902 keV	^89^Y(p,n)^89^Zr	Chelation, commonly with desferrioxamine-based systems	Intact antibodies and slow macromolecules	[^89^Zr]Zr-trastuzumab (investigational)	Long residence increases radiation exposure; high-energy gamma emission and possible *in-vivo* dechelation are relevant
^124^I	4.1760 d	22.6938%; 2137.6 keV	^124^Te(p,n)^124^I on enriched tellurium	Electrophilic radioiodination or prosthetic-group labelling	Proteins/antibodies and iodine-handling pathways	^124^I PET dosimetry and investigational immuno-PET	Complex decay and high positron energy reduce image quality; deiodination and long radiation exposure must be considered

The selected radionuclides illustrate biological time scales from rapid physiology to intact-antibody imaging; the list is not exhaustive. “Biological time-scale fit” denotes a typical use window, not a fixed rule. Half-lives, total beta-plus emission probabilities, and highest beta-plus endpoint energies were extracted from or checked against IAEA LiveChart and the cited IAEA report tables on 9 August 2026. For nuclides with multiple beta-plus branches, the table reports the summed beta-plus probability and the highest listed endpoint energy; these values do not describe one common branch. Values are rounded only for display. Production-route statements are representative. Sources: IAEA Technical Reports Series No. 468 (60); IAEA Radioisotopes and Radiopharmaceuticals Reports No. 1 (61); IAEA-TECDOC-1955 (62); Nelson et al. (9).

### 3.3 Linkers, chelators, and labels are pharmacokinetic variables

The common modular depiction of a radiopharmaceutical—targeting vector, linker, chelator or labelling group, and radionuclide—is useful only if the modules are treated as linked rather than independent. Adding a chelator changes mass, charge, hydrophilicity, coordination geometry, and sometimes protein binding. A linker can separate the binding motif from a bulky radiometal complex, but it can also introduce a metabolic soft spot or alter renal and hepatobiliary clearance. Consequently, a modification that increases uptake at one imaging time point does not necessarily improve time-integrated tumour exposure or tumour-to-organ dose. Figure 2 summarizes these linked molecular modules.

Comparative studies provide stronger design evidence than isolated biodistribution reports. In an early human evaluation of two related FAP-targeting agents, [^68^Ga]Ga-FAPI-04 showed longer tumour retention than [^68^Ga]Ga-FAPI-02, demonstrating that a structural modification can change kinetics while target class and radionuclide remain broadly constant (17). This observation is informative for those constructs but is not a universal rule for all FAP ligands or linkers. The appropriate inference is that linker and scaffold optimization should use head-to-head measurements across multiple time points and report relevant background organs alongside tumour uptake.

Metabolically cleavable designs illustrate the same need for bounded inference. A cleavable linker can reduce normal-organ retention only if cleavage occurs at the intended site and the resulting radiolabelled fragment is stable and rapidly excreted. Cleavage that occurs prematurely in blood or tumour can instead reduce target delivery. The cleavage rate, radiometabolite identity, relevant enzyme distribution and excretion route must therefore be measured for each construct (6). The presence of a cleavable bond is a design hypothesis, not evidence by itself that organ dose is lower.

### 3.4 Affinity, administered mass, and molar activity are coupled

Binding affinity is frequently optimized early because it can be measured reproducibly *in vitro*, yet the highest-affinity ligand is not automatically the best imaging agent. Slow dissociation may increase retention, but high nonspecific binding, poor tissue penetration, rapid metabolism, or delayed background clearance can offset that advantage. Conversely, a modest-affinity ligand may perform well if it reaches accessible targets rapidly and clears efficiently from relevant background tissues. Internalization can support retention for some targets and constructs, but its value depends on the fate of the radiolabel after intracellular processing.

The administered amount of non-radioactive compound adds another source of variation. Molar activity links measured radioactivity to the amount of the corresponding radiolabelled and non-radioactive chemical species. For radiometallated peptides, apparent molar activity may be the operationally relevant quantity because precursor and metal-complexed peptide can remain in the preparation; effective molar activity also accounts for other competitors with chemical, biological, or pharmacological activity (8). In saturable systems, differences in precursor-derived carrier, formulation, or administered mass can change binding, biodistribution, image quality, or pharmacological safety. Reporting activity without chemical amount is therefore insufficient for interpreting a receptor-targeted agent. Molar activity should be defined and measured consistently at a stated reference time using a validated analytical method; it is not a synonym for radiochemical purity or yield.

### 3.5 Diagnostic contrast and therapeutic dose are different endpoints

For diagnostic PET, the operational objective is adequate lesion detectability and quantitative reliability at a clinically practical acquisition time and radiation exposure. A high standardized uptake value is not by itself proof of diagnostic accuracy, because performance also depends on background distribution, lesion size, reconstruction, timing, spectrum of disease, and the reference standard. For therapy, the relevant quantity is closer to time-integrated activity and absorbed dose in tumours and normal tissues. A short-lived peak in tumour uptake can produce an attractive image without providing durable therapeutic exposure.

Early human biodistribution and dosimetry of PSMA-617 illustrate how a defined construct can be characterized before therapeutic efficacy is tested (18). The MIRD schema provides standardized quantities for relating activity over time to absorbed dose, but its application still requires biological data and explicit assumptions about source regions, time-activity curves, geometry, and energy deposition (19). Dosimetry estimates derived from a few imaging time points or from animal-to-human extrapolation should therefore be identified as estimates; they do not directly measure treatment benefit. Comparisons between radionuclides also require more than equal administered activities: differences in emitted energy, branching, half-life, daughter products, and pharmacokinetics affect absorbed dose and biological effect.

### 3.6 A minimum evidence set for design claims

A structure-performance claim should specify the paired constructs, chemical difference, experimental model, administered mass and activity, imaging or sampling times, comparator, and prespecified endpoint. For diagnostic candidates, useful translational endpoints include lesion-level sensitivity and specificity, positive predictive value, reader agreement, quantitative repeatability, and impact on a defined clinical decision. For therapeutic candidates, tumour and normal-organ time-activity data, absorbed-dose estimates, toxicity, and a comparative efficacy endpoint are needed. When only one construct has been studied, the result can establish feasibility or describe biodistribution, but it cannot identify which structural feature caused the observation.

This evidence standard changes how molecular optimization is interpreted. Target affinity, radiochemical yield, a single-time-point tumour-to-background ratio, and an illustrative image are screening measurements. They are not interchangeable with reproducible manufacturing, diagnostic accuracy, organ dosimetry, or clinical benefit. Development should progress only when the next study is designed to resolve the largest remaining uncertainty, instead of accumulating additional measurements of the same favourable property. Table 3 retains direct comparison designs and their interpretive boundaries.

**Table 3:** Direct comparative molecular-design studies and their claim limits.

Design intervention	Paired constructs/comparator	Platform	Observed change under the reported conditions	Evidence setting and question answered	Boundary on interpretation	Primary source
Scaffold/linker optimization for retention	[^68^Ga]Ga-FAPI-02 versus [^68^Ga]Ga-FAPI-04	FAP	FAPI-04 showed longer tumour retention than FAPI-02 while maintaining rapid background clearance in the reported cohort.	Early human biodistribution and dosimetry across heterogeneous cancers	Small, non-randomized study; the comparison does not establish diagnostic accuracy or a universal FAP-linker rule.	Giesel et al. (17)
Administered peptide mass	Low- versus high-peptide-mass [^68^Ga]Ga-ABY-025 with similar activity in the same eight patients	HER2	Kidney and liver absorbed doses were slightly but significantly higher at the low peptide mass; effective dose was 0.030 versus 0.028 mSv/MBq.	Within-patient human biodistribution/dosimetry	Eight patients and a dosimetry endpoint; the study does not define an optimal mass for every HER2 indication or construct.	Sandstrom et al. (44)
Lipophilic amino-acid substitution in a PSMA linker	[^68^Ga]Ga-PSMA-11, [^68^Ga]Ga-PSMA-617, [^68^Ga]Ga-HTK01166 and [^68^Ga]Ga-HTK01167	PSMA	At 1 h in LNCaP-bearing mice, HTK01167 had substantially lower renal uptake than PSMA-617 but also lower tumour uptake; HTK01166 had comparable tumour uptake to PSMA-617 but higher renal uptake.	Head-to-head mouse biodistribution and PET	One xenograft model and a single main time point; improved tumour-to-kidney ratio did not mean increased tumour uptake.	Kuo et al. (63)
Renal brush-border enzyme-cleavable Gly-Tyr linker	Radioiodinated PSMA-769 construct versus a non-cleavable comparator	PSMA	Renal activity was 6–7-fold lower at 21 h, but tumour uptake was also lower (12.6 versus 36.8%ID/g) under the reported comparison.	Paired mouse biodistribution	The favourable tumour-to-kidney ratio arose from changes in both numerator and denominator; radiometal-labelled analogues may behave differently.	Vaidyanathan et al. (64)
Met-Val-Lys renal enzyme-cleavable linker	[^68^Ga]Ga-DOTA-AmBz-MVK(HTK01166) versus the previously reported [^68^Ga]Ga-HTK01166	PSMA	Mean renal uptake was 30% lower at 1 h (104 versus 147%ID/g), but this difference was not statistically significant (*p* = 0.055); the oxidized M(O)VK analogue showed a 39% reduction (*p* = 0.025).	Mouse PET/biodistribution with enzyme and metabolite studies	The comparator came from a previous dataset and the reduction varied with linker oxidation; therapeutic dosimetry was not tested.	Bendre et al. (65)
Tuned albumin binding and PEG8 linker length	Four related ^68^Ga/^177^Lu-labelled RPS ligands, including RPS-063, RPS-071, RPS-072 and RPS-077	PSMA	Stronger albumin binding delayed blood clearance and changed tumour/kidney kinetics; PEG8 weakened albumin binding. RPS-072 achieved the highest reported tumour AUC and tumour-to-kidney AUC ratio in the model.	Head-to-head LNCaP xenograft study	Prolonged blood exposure can increase normal-tissue dose; mouse AUC ranking is not clinical therapeutic benefit.	Kelly et al. (66)

The two human comparisons are listed first, followed by paired animal studies. The clinical/regulatory evidence hierarchy in Section 2 is not applied to a mechanistic structure–performance question for which a controlled paired animal experiment can be the most direct design. Animal rows support construct-specific mechanism or pharmacokinetic hypotheses only and are not ranked as clinical evidence. Human, within-patient, direct animal, and historical-comparator designs remain non-interchangeable. AUC, area under the time-activity curve; PET, positron emission tomography; %ID/g, percentage injected dose per gram.

## 4 Radiochemistry and manufacturability

### 4.1 Design for manufacturing begins before clinical validation

Radiochemistry is often described as the final act of attaching a radionuclide to an optimized ligand. In practice, the labelling route can determine whether the ligand that was optimized is the same chemical entity that can be produced reliably for patients. Precursor synthesis, protecting groups, metal contaminants, reaction solvent, pH, temperature, purification, formulation, container compatibility, and radiolysis can all affect product identity and impurity profiles. A development route that is adequate for a small number of preclinical preparations may be unsuitable for repeated clinical production if it depends on manual handling, poorly characterized precursors, narrow reaction tolerances, or analytical methods that cannot resolve relevant impurities.

Design-for-manufacturing therefore requires an early definition of the intended product. The chemical structure and radionuclide, activity and mass ranges, reference time, formulation, shelf life, route of administration, and critical quality attributes should be stated before a process is optimized. Radiochemical conversion or yield should be reported with an unambiguous definition and measurement point (14). Neither quantity establishes that the formulated drug product has acceptable identity, purity, activity, sterility, or stability.

### 4.2 Radionuclide supply and production scale constrain the process

The production model is radionuclide- and region-dependent. Cyclotron-produced ^18^F can support multi-batch or regional distribution when synthesis, quality control, transport, and administration fit within the decay window. Generator-produced ^68^Ga can support decentralized preparation, while cyclotron production can provide another supply route; the two routes have different starting materials, impurity risks, scale, and regulatory documentation. EANM good-practice recommendations for ^68^Ga accordingly address radionuclide source, metal impurities, labelling, purification, quality control, and facility implementation and do not treat all ^68^Ga chloride as interchangeable (9). Table 2 summarizes selected nuclear and operational properties.

Supply claims should be made at the level of a defined route and setting. “Generator available” does not mean unrestricted batch activity or global access, and “cyclotron produced” does not guarantee economical distribution. Parent radionuclide availability, targetry, enriched material, generator age, elution profile, radionuclidic impurities, transport time, local licensing, and the number of patient doses all affect feasibility. For emerging radionuclides, production reproducibility and a qualified supply chain can be a more immediate barrier than labelling chemistry.

### 4.3 Automation controls execution but does not establish compliance

Automated synthesis can reduce operator exposure, standardize timed operations, and generate electronic or paper batch records. Cassette-based systems can also simplify cleaning validation by replacing product-contact pathways. These benefits are conditional on the validated configuration. A module, cassette, or software sequence is not intrinsically compliant with good manufacturing practice, and the same hardware can produce a nonconforming batch if materials, environmental controls, calibration, process parameters, or documentation are inadequate (20, 21).

Current good radiopharmacy practice for small-scale preparation treats personnel, premises, equipment, materials, documentation, preparation, quality control, release, deviations, and change control as parts of one quality system (22). The process must be qualified over its intended operating range, and changes to a precursor, cassette, reagent supplier, software version, purification step, or analytical method require a documented assessment of product impact. Quality risk management is useful for prioritizing controls, but it does not permit high-risk attributes to be omitted because the product decays rapidly (23).

### 4.4 Product release is attribute- and jurisdiction-specific

Quality-control attributes are sometimes presented as a generic checklist in which appearance, pH, radiochemical identity and purity, radionuclidic identity and purity, chemical impurities, residual solvents, molar activity, sterility, endotoxins, filter integrity, and stability appear to have the same status for every PET radiopharmaceutical. That interpretation is not defensible. The required specification, analytical procedure, sampling plan, and release timing depend on the defined product, applicable pharmacopoeia, marketing or investigational authorization, and jurisdiction.

The general logic is nevertheless consistent. A batch must be shown to meet established criteria for identity, strength or activity, quality, and purity, with sterility and pyrogen control where applicable. US current good manufacturing practice requirements specific to PET drugs are codified in 21 CFR Part 212; 21 CFR 211.1 expressly excludes PET drugs from Part 211 (24, 25). USP <823 > addresses radiopharmaceuticals for PET compounding, investigational and research uses and does not replace product- and jurisdiction-specific specifications (26). For short-lived PET products, bacterial endotoxin and other required rapid tests remain pre-release controls, whereas a compendial sterility result may be completed after release only where the applicable framework permits. Aseptic-process controls and, when used, sterilizing-filter integrity therefore remain critical; a later sterility result does not substitute for them. Table 4 states the timing and caveats, and Figure 4 places these controls within the manufacturing and release pathway.

**Table 4:** Product-specific quality-control attributes and release timing for PET radiopharmaceuticals.

Category	Attribute/test	Typical analytical principle	Release timing	Product-specific caveat	Purpose	Authoritative basis
General	Appearance	Visual inspection under a validated procedure	Before release	Colour/clarity specifications depend on formulation and container; visual inspection cannot replace chemical or particulate tests required by the applicable monograph/label.	Detect visible abnormalities or container-related defects.	21 CFR 212.70; applicable pharmacopoeial/product specification
General	pH	Qualified pH meter, indicator strip or validated microscale method	Before release	The acceptable range is formulation specific; radiation dose and small sample volume can constrain method choice.	Support safety, stability and labelling integrity.	cGRPP (22); applicable pharmacopoeial/product specification
Identity	Radionuclide identity	Half-life measurement and, where informative, energy spectrometry	Before release unless an explicitly permitted route-specific arrangement applies	The common 511-keV annihilation signal does not distinguish positron emitters; half-life and potential contaminants must be considered.	Confirm the intended radionuclide.	Gillings et al. (7), especially radionuclide-identity validation; product specification
Identity	Radiochemical identity	Radio-HPLC or radio-TLC retention comparison with a qualified reference	Before release	Detector delay and matrix effects must be characterized; co-elution can require an orthogonal method.	Confirm the labelled chemical entity.	Gillings et al. (7); 21 CFR 212.70(f) (2)
Purity	Radiochemical purity	Validated radio-HPLC and/or radio-TLC with recovery assessment	Before release	A single method may miss retained or co-eluting species; relevant impurities and decay correction are product specific.	Quantify labelled impurities and intact product fraction.	Gillings et al. (7); 21 CFR 212.70
Purity	Radionuclidic purity	Energy spectrometry and/or decay-corrected delayed measurement	Before release for specified short-lived impurities; delayed testing may be needed for long-lived impurities	Production route determines likely contaminants; a delayed sample can be necessary after decay of the main nuclide.	Control unintended radionuclides and patient/staff dose.	Gillings et al. (7); route-specific IAEA/pharmacopoeial specification
Purity	Residual solvents and other chemical/process impurities	HPLC/UV, mass spectrometry, ion chromatography, GC, colorimetry or other validated method	Before release when required by the specification; timing may be route dependent	Detection limits must be compatible with the specification and small injected mass; methods may not be interchangeable between products.	Control pharmacologically active or process-derived non-radioactive impurities.	Gillings et al. (7); cGRPP (22)
Strength	Activity and activity concentration	Qualified dose calibrator with decay and geometry correction	Before release and at dispensing as required	Calibration setting, container geometry, measurement time and radionuclide purity affect accuracy.	Confirm labelled strength at the reference time and patient dose.	Gillings et al. (7); 21 CFR 212.70
Performance	Molar activity or specific activity, when clinically relevant	Combined activity assay and validated mass quantification	Before release when included in the product specification	It is not universally interchangeable across tracers; the relevant denominator and reference time must be stated.	Control receptor occupancy, mass effects and reproducibility.	Luurtsema et al. (8); 21 CFR 212.70(f) (2)
Microbiology	Bacterial endotoxins	Validated compendial endotoxin test	Before release	Maximum allowable endotoxin is dose- and route-dependent; interference must be evaluated.	Limit pyrogenic risk.	21 CFR 212.70(a); cGRPP (22); applicable pharmacopoeia
Aseptic assurance	Sterilizing-filter integrity, where sterile filtration is used	Validated bubble-point, pressure-hold or equivalent integrity test	As defined by the validated process, normally before administration or release when feasible	This is a process-control test, not a sterility test; method and timing must reflect the filter and closed-system design.	Verify a critical aseptic-processing control.	cGRPP (22); validated process specification
Microbiology	Sterility	Compendial sterility test	May be completed after release for PET drugs under 21 CFR 212.70; testing must start within 30 h after completion of production; weekend or holiday extensions require evidence that delay does not adversely affect the sample or results	A passing later sterility result does not retroactively replace aseptic-process control; failures require documented notification and investigation.	Detect microbial contamination and trigger investigation/corrective action.	21 CFR 212.70(e); cGRPP (22)
Stability	Shelf-life/stability-indicating testing	Time-point activity, pH, appearance, RCP and other product-specific tests	Established during validation; routine testing at the release time point specified	The relevant degradation pathways and container/transport conditions differ by radionuclide and formulation.	Demonstrate that specifications are met through the labelled shelf life.	Gillings et al. (7); cGRPP (22); quality-risk guideline (23)

This table is a decision framework, not a universal release specification. Under 21 CFR 212.70, each PET drug product requires specifications for identity, strength, quality and purity and, when appropriate, sterility and pyrogens. With the stated exception for sterility, conformance must be established before final release. Conditional final release is narrowly limited to analytical-equipment malfunction and cannot be used when radiochemical identity/purity or specific activity cannot be determined. 21 CFR Part 212 is specific to PET drugs; 21 CFR 211.1 excludes PET drugs from Part 211. USP <823 > addresses PET radiopharmaceutical compounding, investigational and research uses (24–26). Jurisdictional law, marketing authorization, pharmacopoeial monographs and the validated site procedure govern actual release.

**Figure 4:**
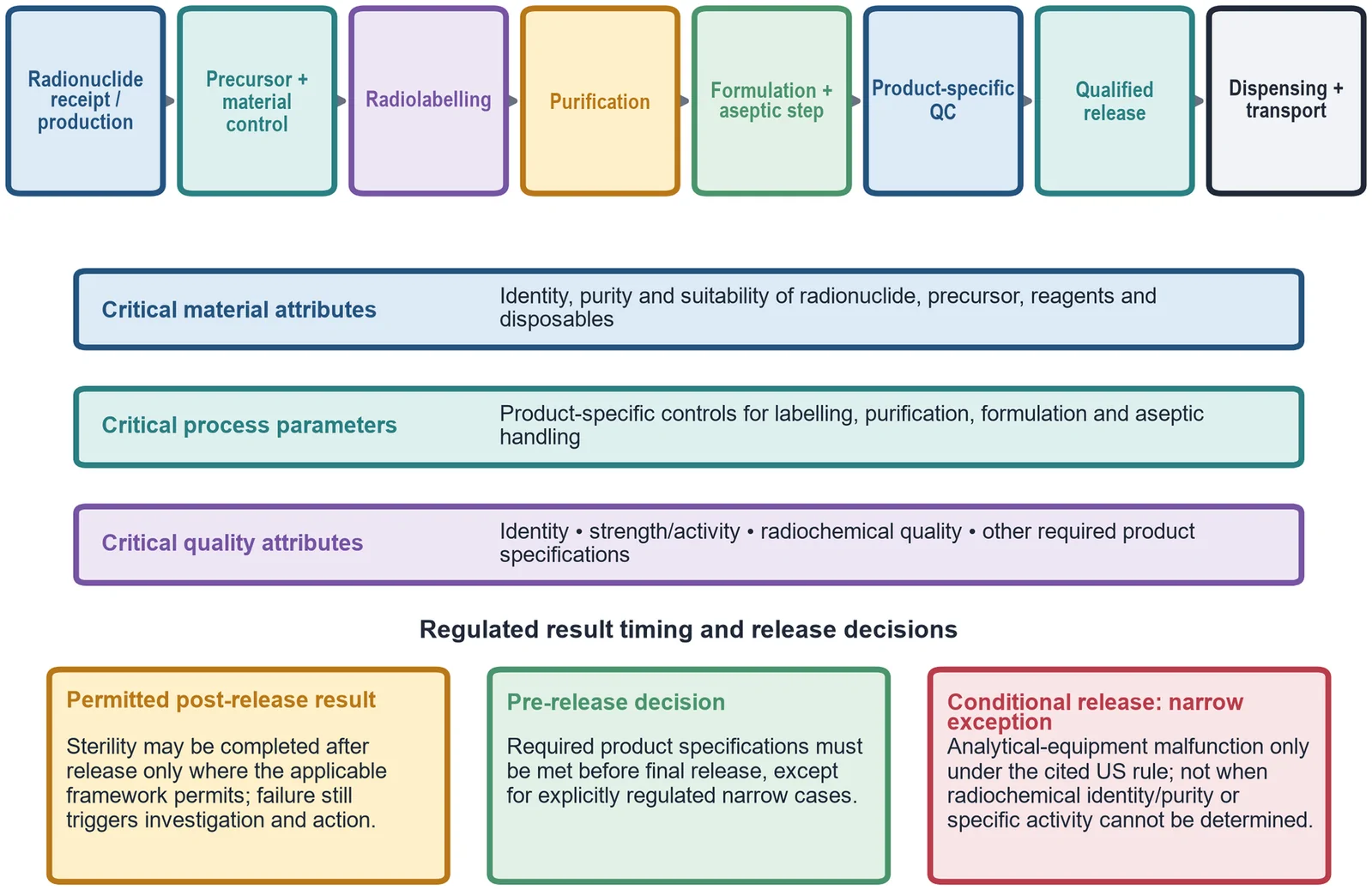
Product-specific manufacturing and release-control pathway. Process map from radionuclide receipt or production through precursor and material control, radiolabelling, purification, formulation and aseptic processing, product-specific quality control, qualified release, dispensing and transport. Critical material attributes, process parameters and quality attributes inform regulated result timing. Bacterial endotoxin and other required rapid tests remain pre-release controls. Sterility may be completed after release only where the applicable framework permits; aseptic-process and filter-integrity controls are not replaced by the later result. The synthesis reflects radiopharmacy guidance, 21 CFR Part 212, USP <823> and the scope statement in 21 CFR 211.1 (7, 8, 21, 24–26).

### 4.5 Analytical methods must be fit for the impurity and time window

An analytical method is useful only if it can distinguish the intended product from relevant radioactive and non-radioactive impurities with adequate specificity, accuracy, precision, range, and robustness. Radio-HPLC and radio-TLC are not interchangeable labels for “purity”; their separation mechanisms, detector response, recovery, sampling, integration, and time required for analysis differ. Identity may require concordance of radioactive and non-radioactive reference signals, while radionuclidic identity and purity depend on decay or emission measurements, not on chromatographic retention alone.

EANM guidance recommends validating analytical methods with attention to the particular constraints of radiopharmaceuticals and provides a framework for selecting characteristics appropriate to the method's intended use (7). Validation must include the formulated product and relevant impurities at concentrations and activities that represent routine batches. A rapid method is not automatically suitable for release if it cannot detect the impurity that defines the safety or performance risk. Conversely, an elaborate research method may be unsuitable if its run time consumes an impractical fraction of the product's shelf life.

Molar activity requires particular care because it combines an activity measurement with chemical quantification and changes continuously with decay. The chemical denominator, activity reference time, calibration, sample dilution, and limit of quantification should be stated. The harmonized EANM guidance also emphasizes that the biological importance of molar activity is target- and mass-dependent, not universal (8). For a saturable receptor system, batch-to-batch mass variation can alter uptake; for another product, the same numerical variation may be clinically irrelevant. A specification should therefore be justified by product knowledge, not copied from an unrelated tracer.

### 4.6 Technology transfer must reproduce the product, not only the script

Multicentre adoption is often presented as a simple transfer of an automated sequence. True comparability requires control of precursor and reagent quality, radionuclide source, contact materials, calibration, environmental conditions, process parameters, purification, formulation, sampling, analytical methods, and reference standards. Two sites can obtain similar activity yields while producing different impurity or mass profiles. Conversely, minor numerical differences in yield can be acceptable when all critical quality attributes remain within justified specifications.

Transfer studies should therefore prespecify which attributes establish comparability and which deviations trigger investigation or revalidation. Batch success rate, synthesis duration, activity yield, radiochemical purity, chemical impurity profile, molar activity, filter integrity, microbiological results, stability, deviations, and out-of-specification events are reportable process outcomes. The translational question is not whether an automated run completed, but whether the defined product can be released reproducibly across time, operators, material lots, and, when relevant, sites (27, 28).

## 5 Clinical validation is platform-specific: mature comparators and emerging targets

### 5.1 Evidence categories must answer different questions

The clinical literature on targeted PET radiopharmaceuticals contains studies of biodistribution, dosimetry, lesion detection, diagnostic accuracy, management impact, treatment response, toxicity, and survival. These endpoints should not be collapsed into a single label such as “clinically validated.” A first-in-human study can show that a product was administered and describe where radioactivity was observed. A diagnostic-accuracy study can estimate performance against a reference standard in a defined population. A randomized therapeutic trial can test whether treatment changes an outcome relative to a comparator. Regulatory authorization then defines a product, indication, jurisdiction, and date; it is not a permanent global property of a molecular target.

This distinction separates two established radiotheranostic platforms, PSMA in prostate cancer and somatostatin receptors in neuroendocrine tumours, from FAP/FAPI and other targets that have substantial human imaging experience but less mature therapeutic evidence. “Established” does not mean free of false-positive uptake, target-negative disease, treatment toxicity, or implementation barriers. Here it means that a platform includes a reproducible selection method, randomized therapeutic evidence, and at least one region-specific authorization for a defined population. Table 1 compares representative platform maturity.

### 5.2 PSMA: strong clinical evidence with selection and interpretation constraints

PSMA-targeted PET has been evaluated in prospective multicentre studies across biochemical recurrence and initial staging. In a prospective study of 635 men with biochemical recurrence, [^68^Ga]Ga-PSMA-11 PET showed a high positive predictive value among lesions that could be validated and substantial inter-reader agreement, although the reference standard was not uniform for every detected lesion (29). OSPREY evaluated [^18^F]DCFPyL in high-risk primary disease and suspected recurrent or metastatic disease. In the surgical cohort, specificity for pelvic nodal disease was high, but sensitivity was approximately 40% and did not meet the prespecified sensitivity endpoint (30). These results show why a positive scan and a negative scan have different evidentiary meanings: high specificity can support localization of a positive lesion, whereas limited sensitivity means that microscopic nodal disease cannot be excluded by a negative scan.

Tracer chemistry and biodistribution also influence interpretation. [^18^F]PSMA-1007 provides logistical and urinary-clearance features that may be useful in selected settings, but multicentre retrospective work has documented focal unspecific bone uptake as a potential pitfall (31). The problem is not resolved by a universal standardized uptake value threshold because benign and malignant uptake can overlap and interpretation depends on location, morphology, disease context, and other lesions. Similar caution applies to PSMA uptake outside prostate cancer and to physiologic or benign uptake in normal tissues. A review should therefore identify the exact agent when discussing performance instead of treating “PSMA PET” as a chemically homogeneous test (32).

The therapeutic evidence is strongest for [^177^Lu]Lu-PSMA-617. VISION enrolled participants with progressive, PSMA-positive metastatic castration-resistant prostate cancer after prior use of an androgen-receptor pathway inhibitor and exposure to either one or two taxane regimens. Adding [^177^Lu]Lu-PSMA-617 to protocol-permitted standard care improved imaging-based progression-free survival (median 8.7 versus 3.4 months; hazard ratio 0.40) and overall survival (median 15.3 versus 11.3 months; hazard ratio 0.62), while grade 3 or higher adverse events were more frequent in the radioligand-therapy group (2). These results support benefit in the studied population; they do not establish the same benefit in earlier disease, in patients who fail the trial's imaging criteria, or for other PSMA ligands and radionuclides.

PSMAfore was a phase 3 randomized trial in taxane-naive patients with PSMA-positive metastatic castration-resistant disease that had progressed on one androgen-receptor pathway inhibitor. Median radiographic progression-free survival was 9.3 months with [^177^Lu]Lu-PSMA-617 and 5.6 months with an alternative androgen-receptor pathway inhibitor (hazard ratio 0.41) (4). Overall survival was not statistically different at the FDA analysis; interpretation was complicated by protocol-permitted crossover, with 57% of control-arm patients crossing over to [^177^Lu]Lu-PSMA-617 in the primary publication (4). The phase 2 TheraP trial provides a separate randomized comparison with cabazitaxel in a post-taxane population (33). The US indication was expanded on 28 March 2025 to adults previously treated with an androgen-receptor pathway inhibitor who were considered appropriate to delay taxane-based chemotherapy. At the review date, the EMA description remained different. Figure 5 and Table 1 therefore display jurisdiction and verification date rather than one undifferentiated ‘approved’ status (10, 11).

**Figure 5:**
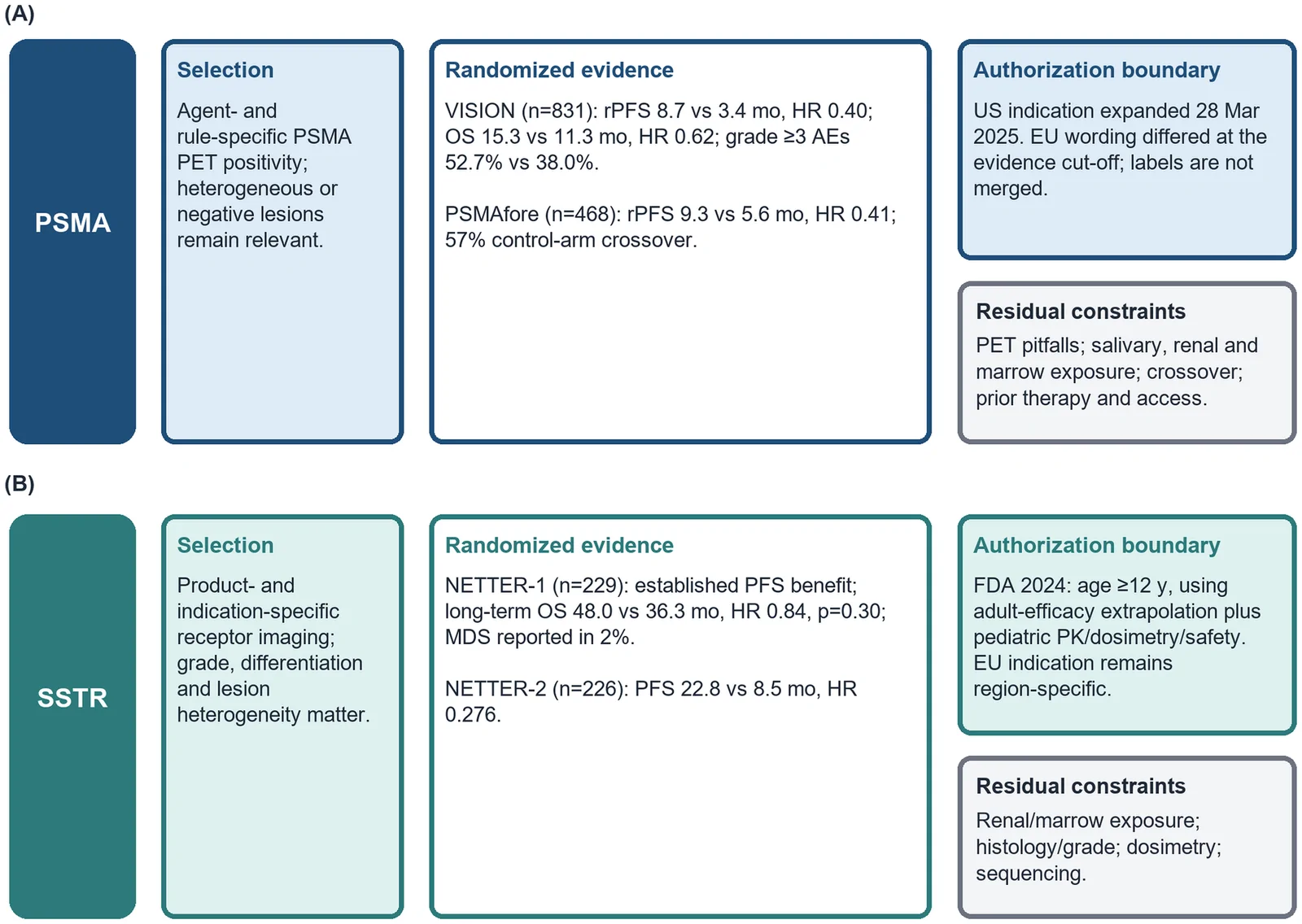
What makes a radiotheranostic platform clinically established. Parallel evidence chains show why neither imaging positivity nor biological plausibility alone establishes a radiotheranostic platform. **(A)** PSMA: product- and rule-specific PET selection is linked to VISION (2) and PSMAfore (4) randomized evidence and to date- and region-specific authorization sources (10, 11, 56). **(B)** SSTR: product- and indication-specific receptor imaging is linked to NETTER-1 (3, 34) and NETTER-2 (5) randomized evidence and to the US paediatric extension and European product information (35, 59). Side boxes retain residual constraints involving target-negative disease, uptake pitfalls, organ exposure, crossover, histology/grade, dosimetry, sequencing and access. Numerical values are reproduced only from the cited primary publications or regulator pages; regional labels are not merged.

Three lessons follow from the PSMA platform. First, selection is product- and rule-specific: the diagnostic agent and interpretation criteria used for eligibility must be stated. Second, an imaging biomarker can enrich a treatment population without being a perfect measure of radiosensitivity or absorbed tumour dose. Third, a platform supported by randomized trials still requires attention to heterogeneous expression, discordant lesions, marrow and renal safety, normal-organ uptake, prior therapies, and access to both imaging and treatment.

### 5.3 Somatostatin receptors: a mature platform with evolving treatment settings

Somatostatin receptor imaging and peptide receptor radionuclide therapy provide the other major example of clinically integrated nuclear theranostics. Radiolabelled somatostatin analogues allow whole-body assessment of receptor-positive disease, while [^177^Lu]Lu-DOTA-TATE delivers therapy to selected patients. As with PSMA, the platform is not a single interchangeable pair. DOTATATE, DOTATOC, DOTANOC, receptor agonists and antagonists differ in receptor profile, affinity, internalization, clearance, approved status, and the evidence available for a particular use.

NETTER-1 was a phase 3 randomized trial of patients with advanced, well-differentiated, somatostatin receptor-positive midgut neuroendocrine tumours that had progressed during standard-dose long-acting octreotide. [^177^Lu]Lu-DOTA-TATE plus long-acting octreotide produced a marked progression-free-survival benefit over high-dose long-acting octreotide in the primary analysis (3). In the prespecified long-term analysis, median overall survival was 48.0 versus 36.3 months, but the difference was not statistically significant (hazard ratio 0.84; *p* = 0.30) (34). Both results belong in a balanced review: the trial established progression-free-survival benefit and supported clinical adoption, while the final overall-survival endpoint did not meet statistical significance.

NETTER-2 was an open-label phase 3 randomized trial of first-line [^177^Lu]Lu-DOTA-TATE plus long-acting octreotide in newly diagnosed, advanced, well-differentiated grade 2–3 gastroenteropancreatic neuroendocrine tumours. Median progression-free survival was 22.8 months with [^177^Lu]Lu-DOTA-TATE and 8.5 months with high-dose octreotide (hazard ratio 0.276) (5). This extends the evidence to an earlier and biologically distinct population, but it should not be generalized to poorly differentiated neuroendocrine carcinoma or to all grades and primary sites. Histology, grade, pace of disease, receptor distribution, competing systemic options, renal and marrow reserve, and the pattern of lesions remain necessary clinical variables.

Regulatory development also illustrates the difference between direct trial evidence and extrapolation. In April 2024, the FDA approved [^177^Lu]Lu-DOTA-TATE for patients aged 12 years and older with somatostatin receptor-positive gastroenteropancreatic neuroendocrine tumours. The paediatric decision used pharmacokinetic, dosimetry, and safety data from nine paediatric patients together with extrapolation of adult efficacy; no paediatric randomized efficacy trial was performed (35). The approval is real, but the evidence basis should be represented accurately in a review and evidence-maturity figure.

The remaining uncertainties are not arguments against the platform. They define the next comparative questions: when receptor imaging should be complemented by metabolic imaging; how lesion-level heterogeneity should alter sequencing; whether individualized dosimetry improves outcomes over fixed-activity regimens in specific populations; and how retreatment, combinations, and earlier-line use affect cumulative marrow and renal risk. These questions require prospective endpoints rather than a generic statement that “personalization is the future.”

### 5.4 FAP/FAPI: broad human imaging experience, immature therapeutic evidence

FAP-targeted imaging differs biologically from tumour-cell receptor imaging because many agents primarily interrogate activated fibroblasts and stromal remodelling. This can produce uptake across diverse malignancies, but it also means that uptake is not synonymous with a cancer-cell-specific target. The clinical meaning depends on tumour type, stromal composition, fibrosis, inflammation, wound repair, prior treatment, and the kinetic behaviour of the individual ligand.

Early human studies established feasibility and heterogeneous uptake, not universal diagnostic accuracy. Giesel and colleagues compared related [^68^Ga]Ga-FAPI constructs and reported rapid targeting with differences in tumour retention (17). Kratochwil and colleagues retrospectively described [^68^Ga]Ga-FAPI uptake in 80 patients across 28 cancer types; uptake varied substantially between and within tumour categories, and the study was not designed to establish sensitivity, specificity, or patient-outcome benefit for a single indication (36). These data justify disease-specific prospective evaluation. They do not justify calling FAPI a pan-cancer replacement for [^18^F]FDG (37).

Therapeutic studies remain earlier in development. A first-in-human report of [^177^Lu]Lu-FAP-2286 included 11 patients with advanced adenocarcinomas after confirmation of FAP-targeted PET uptake and focused on feasibility, biodistribution, and preliminary dosimetry (38). A dose-escalation study of albumin-binder-conjugated [^177^Lu]Lu-EB-FAPI in metastatic radioiodine-refractory thyroid cancer included 12 patients and reported response and safety observations without a randomized comparator (39). Both studies generate dose, retention, safety, and trial-design hypotheses. Their small samples, selected populations, heterogeneous prior treatment, and lack of a control group preclude confirmatory claims about comparative efficacy. Related studies of a dimeric FAPI construct and FAPI-74 provide construct-specific imaging and dosimetry evidence, not comparative therapeutic efficacy (40, 41).

No FDA or EMA authorization for a FAP/FAPI radiopharmaceutical was identified in the official pages searched for this Review as of 9 August 2026. This statement is deliberately narrower than “not approved worldwide,” because a global regulatory-database review was not performed. Figure 6 therefore displays FAP/FAPI as a platform with human imaging, dosimetry, and early therapeutic studies but without randomized comparative therapeutic evidence identified in the present search.

**Figure 6:**
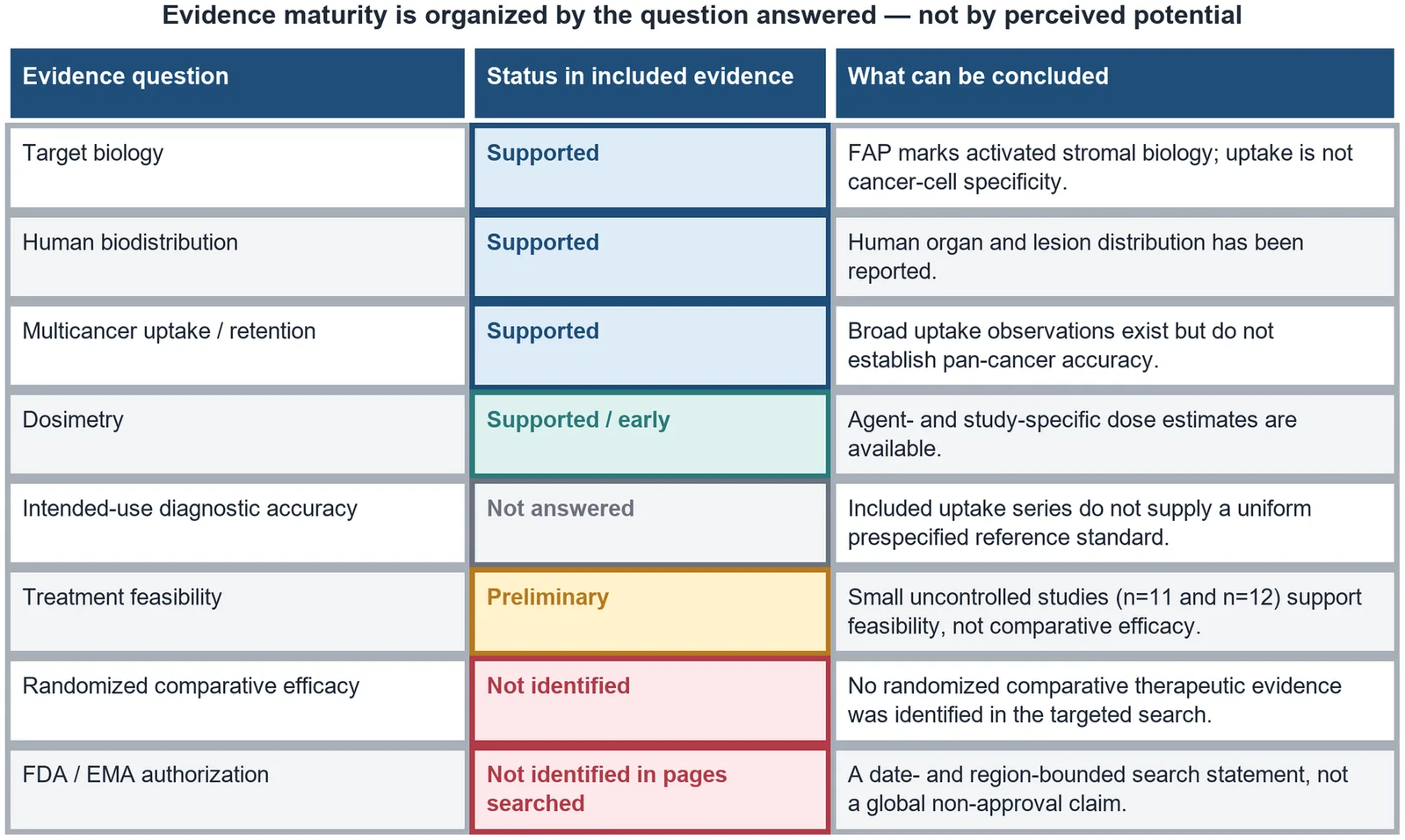
FAP-targeted radiopharmaceuticals: evidence maturity by question answered. Evidence matrix for FAP/FAPI divided into target biology, human biodistribution, multicancer uptake/retention, dosimetry, intended-use diagnostic accuracy, treatment feasibility, randomized comparative efficacy, and authorization. Status labels report only what the included evidence answers; an unanswered cell is not evidence of biological failure. Human imaging and dosimetry reports (17, 36) and small uncontrolled treatment studies (38, 39) are separated from randomized comparative therapeutic evidence. The FDA/EMA authorization row records the bounded official-page search through 9 August 2026 rather than a permanent global claim. Broad uptake observations therefore cannot be read as pan-cancer diagnostic accuracy or therapeutic benefit.

### 5.5 What can and cannot be transferred across platforms

PSMA, somatostatin receptor, and FAP-targeted agents share a modular radiopharmaceutical vocabulary, but their evidence cannot be transferred by analogy. An imaging-positive threshold validated for one target does not define eligibility for another. A radionuclide and chelator that are well established with one vector do not guarantee stability or organ dose with a different vector. A fixed administered activity supported by one product's trials does not validate the same schedule for a chemically related ligand.

The transferable element is the evaluation logic: define the chemical product; demonstrate target-relevant biodistribution; characterize mass effects, metabolism, and critical-organ exposure; validate production and analytical methods; test diagnostic or therapeutic performance against an appropriate comparator; and state the population and jurisdiction to which the conclusion applies. This logic supports innovation while preventing early feasibility data from being presented as established clinical benefit.

## 6 Edge cases that expose design constraints

The number of reported PET targets is too large for a useful catalogue, and publication of a first human image is not a sufficient inclusion criterion for a next-generation platform. The most informative examples are those that expose a design problem not already captured by PSMA, somatostatin receptors, or FAP. Human epidermal growth factor receptor 2 (HER2)-targeted PET shows how vector size and administered protein mass alter imaging. C-X-C chemokine receptor type 4 (CXCR4)-directed imaging and therapy show how target expression in haematological tissues, marrow toxicity, and treatment sequencing can dominate the theranostic design.

### 6.1 HER2-targeted PET: antibody kinetics versus engineered proteins

[^89^Zr]Zr-trastuzumab uses the biological specificity of an intact therapeutic antibody and a radionuclide with a physical half-life compatible with delayed immunoPET. Early human work established biodistribution and the ability to visualize HER2-positive lesions (42). A small prospective study subsequently used [^89^Zr]Zr-trastuzumab PET to identify HER2-positive metastases in patients whose primary breast cancers had been classified as HER2-negative; biopsy examined selected PET-positive lesions (43). The scientific value is whole-body assessment of spatial heterogeneity. Slow blood clearance, delayed imaging, radiation exposure, prior trastuzumab, and administered antibody mass can influence lesion contrast. This example stresses the vector-timescale and administered-mass gates rather than supporting a general preference for antibodies or engineered proteins.

Engineered HER2-binding proteins provide a different compromise. [^68^Ga]Ga-ABY-025, an Affibody molecule, was evaluated at two peptide mass doses in eight patients. Kidney and liver uptake contributed substantially to dosimetry, and measured distribution differed with administered mass (44). This study is especially relevant to radiopharmaceutical design because it shows that a “microdose” is not a chemically neutral detail. Changing the non-radioactive mass can modify target and normal-tissue binding even when administered activity is similar. The correct development question is therefore not whether antibodies or small proteins are superior in general, but which vector and mass range produce a reproducible relationship between HER2 status, lesion uptake, background clearance, and the intended clinical decision.

### 6.2 CXCR4: target heterogeneity and marrow risk

[^68^Ga]Ga-pentixafor has been studied as an imaging agent for CXCR4 expression, particularly in haematological malignancies. In a 34-patient comparison in treatment-naive multiple myeloma, [^68^Ga]Ga-pentixafor and [^18^F]FDG showed discordant disease extent in a substantial fraction of patients, illustrating that the two tracers measure different biological features and are not interchangeable staging tests (45). A larger or brighter lesion set on one tracer does not by itself establish superior outcome prediction or management benefit; lesion validation, reproducibility, and prospective decision-impact studies remain necessary.

The therapeutic counterpart, labelled pentixather, also illustrates why imaging uptake cannot be separated from organ biology. The first-in-human report included three heavily pretreated patients who underwent CXCR4-directed endoradiotherapy followed by chemotherapy and autologous stem-cell transplantation for marrow rescue (46). This was a feasibility experience in an intensive multimodality treatment sequence, not evidence for stand-alone radioligand efficacy. Target expression in marrow and other normal haematopoietic compartments, pretherapeutic dosimetry, conditioning, rescue strategy, and patient selection are central to the therapeutic concept.

### 6.3 Criteria for adding an emerging platform to clinical comparison

An emerging target should enter the main evidence table only when the review can identify a defined radiopharmaceutical, a human population, a study design, a measurable endpoint, and a clinically relevant limitation. Platforms supported only by cell binding or xenograft images belong in a preclinical evidence inventory, not beside products with human comparative evidence or authorization. For diagnostic agents, priority should be given to prospective comparisons with pathology, an accepted imaging standard, or a prespecified management endpoint. For therapeutic agents, absorbed-dose characterization and controlled clinical outcomes are required before “theranostic” is used to imply benefit rather than chemical pairing. CXCR4 particularly stresses the normal-tissue exposure, marrow-risk, and implementation gates because imaging, conditioning, and stem-cell rescue form one treatment sequence. Table 5 maps failure modes to evidence that can reduce the relevant uncertainty.

**Table 5:** Translational failure modes and studies that can reduce the relevant uncertainty.

Translational failure mode	Observable consequence	Minimum evidence needed	Suitable study/validation design	Decision-relevant endpoint	Residual limitation after a positive result
Target expression is heterogeneous, inaccessible or temporally unstable	Target-negative lesions, discordance with pathology, or changing uptake after therapy	Spatially and temporally matched imaging-tissue evidence plus lesion-level follow-up	Prospective multi-lesion cohort with prespecified pathology or validated clinical reference standard	Lesion-level detection/discordance by disease site and prior treatment	Biopsy sampling and temporal mismatch remain; a negative scan can reflect biology or technical sensitivity.
High initial uptake is not retained	Late loss of tumour activity and reduced therapeutic residence time	Serial quantitative imaging and metabolite-aware time-activity data	Head-to-head construct comparison over a biologically relevant interval	Tumour AUC, effective half-life and tumour-to-organ AUC ratios	Imaging time points may be sparse and small-animal kinetics may not scale to humans.
Normal-organ exposure rises with tumour delivery	Kidney, salivary gland, marrow, liver or blood dose becomes dose limiting	Patient-specific or population time-integrated organ and tumour dosimetry	Serial quantitative SPECT/PET with harmonized calibration and organ segmentation	Absorbed dose distributions and exposure-response/toxicity associations	Dosimetry precision does not by itself establish that dose adaptation improves outcomes.
Administered mass or molar activity changes binding	Batch- or dose-dependent uptake and background distribution	Mass-range and activity-range characterization	Within-subject imaging comparison where ethical, or prospective dose/mass escalation	Uptake, occupancy-sensitive organs, lesion contrast and safety by mass/activity	An optimum for one construct or population may not transfer to another.
Label or chelate is unstable *in vivo*	Bone, liver, thyroid or other radiometabolite uptake; discordant late images	Plasma/urine metabolite analysis plus challenge and biodistribution studies	Orthogonal *in-vitro* stability, animal metabolite studies and first-in-human serial sampling	Fraction intact, identity of radiometabolites and organ-specific accumulation	Species differences and low-mass analytical sensitivity can obscure rare metabolites.
Radiosynthesis is not reproducible across runs or sites	Variable yield, RCP, mass, impurity profile or failed batches	Defined critical material/process attributes and multi-batch reproducibility	Prospective process validation and risk-based technology transfer	Batch success rate and distributions of validated critical quality attributes	Validated performance can change after material, module or scale changes and requires change control.
An analytical method is not fit for the impurity or decay window	False pass/fail, unmeasured retained species or late release	Specificity, recovery, sensitivity, precision and range under actual radioactive conditions	Product-specific method validation/verification with spikes and orthogonal methods	Ability to quantify each relevant impurity at its limit within the available time	Reference impurities may be unavailable, and radiation/detector geometry can remain method specific.
Diagnostic performance is inferred from uptake alone	Attractive images without reliable sensitivity, specificity or management value	Prespecified reference standard and blinded interpretation	Prospective multicentre diagnostic-accuracy study in the intended-use population	Patient- and lesion-level sensitivity/specificity, indeterminate rate and management impact	Accuracy may not translate into improved patient outcomes or cost-effectiveness.
Therapeutic efficacy is inferred from dosimetry or uncontrolled response	Apparent response confounded by selection, prior therapy or natural history	Comparative clinical outcome evidence and transparent eligibility criteria	Randomized trial, or a justified prospective comparative design when randomization is infeasible	Progression-free/overall survival, validated response, patient-reported outcomes and grade ≥3 toxicity	Crossover, subsequent therapy and evolving standards of care can dilute or complicate inference.
Imaging response is used as an unvalidated surrogate	Early uptake change is overinterpreted as survival benefit	Reproducibility and outcome-association data for an agent- and disease-specific framework	Prospective response study with independent review and longitudinal clinical outcomes	Measurement repeatability, categorical agreement and association with established outcomes	Consensus criteria can improve consistency without proving surrogacy.
Regulatory or supply assumptions are transferred across regions	A product is described as available or approved where it is not; clinical implementation fails	Date-stamped official authorization and supply-chain verification	Jurisdiction-specific implementation assessment	Authorized indication, product availability, validated transport/shelf life and service capacity	Authorizations, labels, reimbursement and supply can change after publication.

This table is an evidence-planning synthesis by the authors, grounded in the analytical-validation, quality-risk, dosimetry, procedure-guideline and clinical-trial sources cited in the Review (2–9, 19, 22–24, 47–50, 52, 55). A “minimum” item reduces one uncertainty; it is not sufficient by itself for authorization or clinical adoption.

This criterion intentionally excludes many attractive molecular targets from detailed treatment. It prevents the review from becoming obsolete as new probes appear and keeps attention on generalizable design lessons: match vector kinetics to radionuclide and acquisition time; measure administered mass effects; account for normal-tissue target expression; establish whether whole-body imaging adds information beyond tissue sampling; and do not infer therapeutic benefit from diagnostic uptake alone.

## 7 Closing the theranostic loop without overclaiming

Precision theranostics is often illustrated as a diagnostic scan followed by a matched treatment. That sequence is incomplete. A clinically useful theranostic pathway is a feedback loop in which eligibility, treatment delivery, dosimetry, toxicity, and response are reassessed against the same product-specific evidence standard. The loop is only as reliable as its weakest link. High uptake cannot compensate for an ineligible disease setting, an unvalidated interpretation rule, inadequate organ reserve, or a treatment product whose pharmacokinetics differ materially from the diagnostic agent.

### 7.1 Eligibility is an agent- and indication-specific decision

Patient selection begins with histology, disease state, prior treatment, performance status, organ function, and the therapeutic indication. Targeted PET adds whole-body information on target availability and lesion heterogeneity, but it does not replace these clinical variables. For PSMA-directed therapy, procedure guidance requires the imaging agent, acquisition conditions, interpretation criteria, relevant normal-organ uptake, and sites of discordant disease to be considered together (47, 48). The eligibility rule used in a randomized trial should not be silently replaced by a different tracer, threshold, or reading method and then described as trial-equivalent.

The same principle applies to somatostatin receptor-directed therapy. Receptor-positive disease on PET is necessary for the populations represented in the pivotal trials, but uptake must be interpreted with tumour grade, disease tempo, lesion distribution, renal and marrow reserve, and alternative treatments. In both platforms, a patient-level label can conceal lesion-level heterogeneity. A clinically meaningful report should therefore identify whether substantial disease lacks the target phenotype, rather than reducing the scan to a binary positive or negative result.

### 7.2 Treatment delivery must remain product specific

The treatment step should follow the authorized label, applicable national regulation, and product-specific professional guidance. This Review does not propose generic administered activities, cycle numbers, intervals, or organ-dose limits. Those parameters depend on the radiopharmaceutical, indication, jurisdiction, patient characteristics, concomitant care, and the evidence supporting the regimen. Chemically related agents should not be assumed to share the same activity schedule or toxicity profile.

Verification of delivery includes more than recording the dispensed activity. It requires confirmation of product identity and release status, administered activity and time, relevant deviations, and post-administration observations. Where quantitative post-therapy imaging is used, camera calibration, acquisition timing, reconstruction, segmentation, and uncertainty reporting become part of the clinical measurement. A technically successful administration is not equivalent to adequate tumour irradiation, and a high lesion count rate is not itself a treatment-response endpoint.

### 7.3 Dosimetry is a measurement framework, not proof of benefit

Patient-specific dosimetry can describe how activity changes over time and estimate absorbed dose in tumours and normal organs. EANM recommendations for [^177^Lu]Lu-labelled somatostatin receptor and PSMA ligands specify practical requirements for quantitative imaging, time-activity integration, mass or volume definition, absorbed-dose calculation, and uncertainty assessment (49). Joint EANM/MIRD guidance similarly emphasizes calibration and quantitative SPECT methods for ^177^Lu applications (50). These standards make dosimetry more reproducible; they do not remove biological uncertainty or establish that any particular dose-based prescription improves survival (51).

This distinction is important because three questions are often merged. The first is whether patient-specific absorbed dose can be estimated with acceptable uncertainty. The second is whether absorbed dose correlates with tumour control or toxicity in a defined population. The third is whether changing treatment on the basis of that estimate improves clinical outcomes compared with the tested activity-based regimen. The EANM position paper on European radiation-protection requirements explicitly noted that superiority of patient-specific dosimetry-based prescription had not been established, while encouraging research toward dose-guided planning (52). A balanced review should therefore support dosimetry as a tool for measurement, learning, and prospective testing without claiming an unproven outcome advantage (53, 54).

### 7.4 Response requires multimodal reassessment

Post-treatment assessment should integrate symptoms, adverse events, laboratory measurements, anatomical imaging, relevant biochemical markers, and molecular imaging when it addresses a defined question. Changes in tracer uptake may reflect target modulation, treatment effect, tumour-cell loss, inflammation, timing, reconstruction, or administered mass. Uptake alone cannot distinguish all of these mechanisms. New lesions, target-negative progression, and discordance between molecular and anatomical findings must be evaluated explicitly.

The recently proposed somatostatin receptor PET response framework illustrates both progress and the remaining evidence gap. It uses changes in receptor-expressing tumour volume and new lesions; standardized uptake value alone is insufficient, but it was developed by expert consensus and had not yet been validated against survival outcomes at publication (55). It should therefore be described as a harmonization framework for prospective validation, not as a proven surrogate endpoint. Comparable product- and disease-specific response standards remain necessary for other theranostic platforms.

### 7.5 Implementation determines whether the loop is usable

Even a biologically coherent pathway can fail if isotope supply, production capacity, qualified staff, scanner access, dosimetry resources, radiation protection, waste management, or multidisciplinary coordination are inadequate. These constraints are not merely administrative. Delayed imaging, altered production routes, decentralized versus on-site preparation, and limited access to treatment can change which patients are assessed and how closely practice matches trial conditions.

The most defensible clinical loop therefore records five linked decisions: why the patient is eligible; which imaging product and interpretation rule were used; which therapeutic product and regimen were delivered; how toxicity and, when applicable, absorbed dose were assessed; and what evidence triggered continuation, modification, or discontinuation. Figure 7 shows these decision points separately from the platform-evidence chains in Figure 5. It does not imply a universal pathway or a generic dose-escalation algorithm.

**Figure 7:**
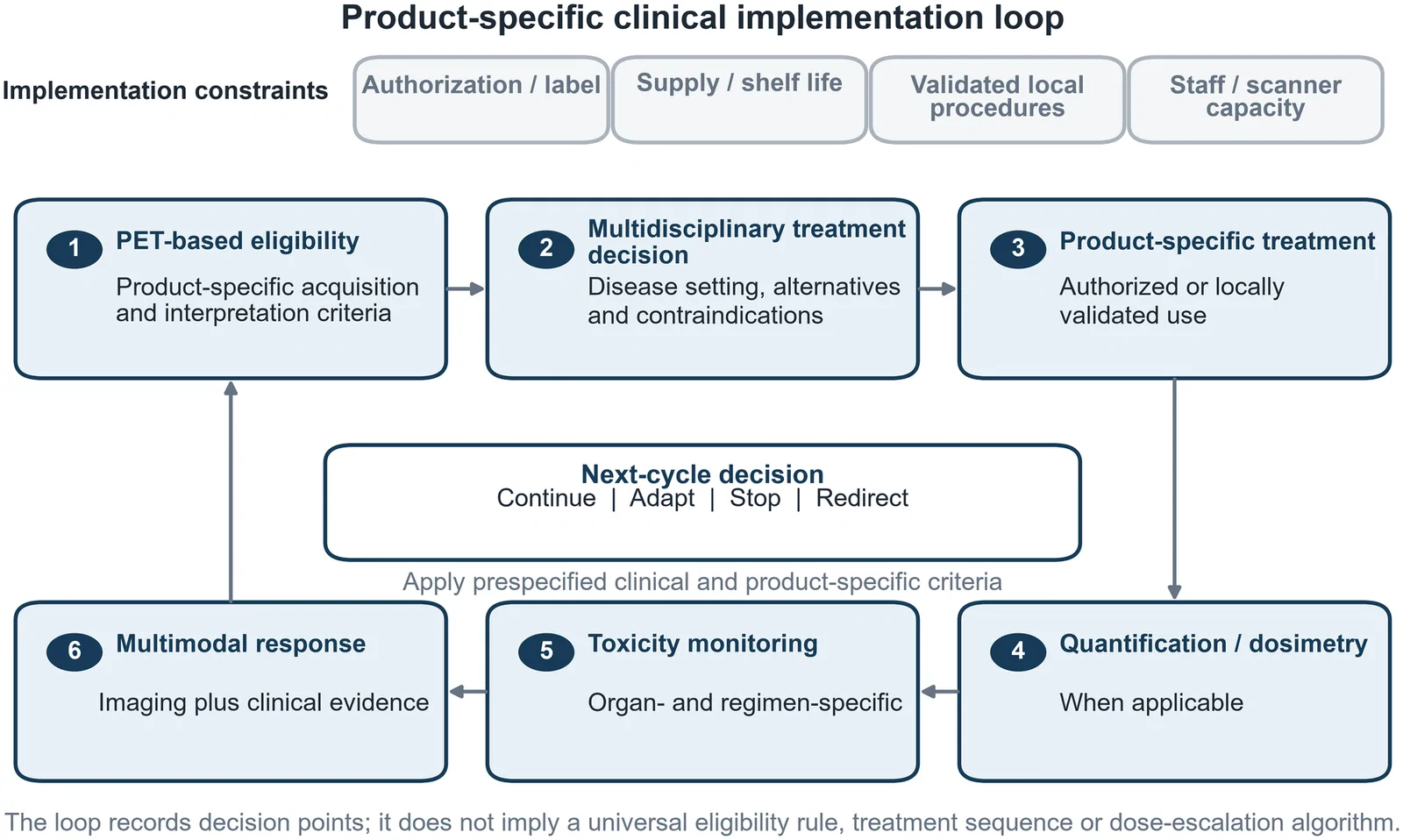
Product-specific clinical implementation loop. Closed-loop framework linking PET-based eligibility using product-specific acquisition and interpretation criteria, multidisciplinary treatment decisions incorporating disease setting, alternatives and contraindications, product-specific treatment, quantitative imaging or dosimetry where applicable, toxicity monitoring and multimodal response assessment. The central annotation lists next-cycle options (continue, adapt, stop or redirect) that require prespecified clinical and product-specific criteria. The loop records decision points and is not a universal eligibility rule, treatment sequence or dose-escalation algorithm.

## 8 Discussion

The central argument of this Review is that PET radiopharmaceutical translation is an exposure-control problem constrained by evidence. A useful construct must place sufficient radioactivity at the intended biological target on the relevant time scale, limit exposure of critical normal tissues, remain chemically defined during production and use, and answer a clinical question with a validated interpretation. No single laboratory metric captures this system. Affinity, radiochemical yield, a visually striking scan, or even target-positive human biodistribution can each support one part of the argument, but none establishes clinical utility alone.

This view changes how molecular optimization should be judged. The common preference for ever higher affinity is rational only when stronger binding improves the endpoint that matters. For a rapidly clearing diagnostic ligand, higher affinity may improve retention and contrast. It may also increase binding-site effects, slow background clearance, or magnify uptake in a normal organ that expresses the target. For a therapeutic agent, peak uptake is even less informative because tumour and organ dose depend on the time-integrated distribution of the labelled species and its radiometabolites. Head-to-head comparisons of paired constructs, with administered mass, imaging times, metabolites, and relevant organs controlled, are therefore more informative than comparisons assembled across unrelated studies.

The evidence from mature theranostic platforms also places diagnostic uptake in context. PSMA and somatostatin receptor imaging can enrich populations for targeted radionuclide therapy, but randomized benefit was established for specific products, disease settings, selection rules, and regimens. The result is not a universal theorem that any target-positive scan predicts benefit from any matched therapeutic compound. VISION, PSMAfore, NETTER-1, and NETTER-2 each answer a bounded clinical question. Differences in prior treatment, grade, comparator, crossover, diagnostic product, and regulatory indication limit cross-trial extrapolation. Evidence-maturity figures and tables should preserve these boundaries instead of placing every platform on an apparently continuous path toward approval.

FAP-targeted agents reveal the opposite risk. Broad uptake across tumour types and rapid publication of human images can create the impression of a nearly universal platform. The biological signal, however, often originates in activated fibroblasts and stromal remodelling rather than a cancer-cell-specific antigen. Uptake can occur in inflammation, fibrosis, wound repair, and treatment-related change, while tumour retention varies by compound and disease. Early treatment studies of FAP-targeted agents have generated useful pharmacokinetic, dosimetric, and safety hypotheses, but small uncontrolled cohorts cannot establish comparative efficacy. The correct next step is not another pan-cancer image atlas. It is disease- and product-specific testing with explicit reference standards, prospective endpoints, and a comparator appropriate to the intended clinical use.

Manufacturing adds a second layer of non-transferability. Automation can reduce operator-dependent variation, but it does not establish that different modules, cartridges, precursor lots, purification methods, or starting radionuclides produce an equivalent drug product. Radiochemical conversion, yield, purity, identity, chemical impurities, molar activity, sterility-related controls, and stability answer different questions. A release table that assigns one acceptance limit to all PET radiopharmaceuticals would be misleading. Product specifications must instead follow the validated process, applicable pharmacopoeial or regulatory requirements, and the risk posed by tests that are completed only after administration.

Dosimetry occupies a productive but contested position between measurement and treatment optimization. Quantitative imaging and standardized calculation methods can reveal large inter-patient and inter-organ differences that are hidden by administered activity alone. These measurements can support safety assessment, explain unexpected toxicity or poor tumour exposure, and generate hypotheses for treatment adaptation. Yet a technically credible absorbed-dose estimate does not itself prove that dose-guided prescription improves outcomes. That claim requires prospective comparison against the regimen supported by current trials, including toxicity, tumour control, quality of life, and resource burden. The review therefore rejects both extremes: dosimetry should not be dismissed as optional research, but its clinical superiority should not be assumed before it is tested.

Regulation and access further complicate the idea of a single translational pathway. Authorization is attached to a product, indication, population, and jurisdiction, and it can change over time. The 2025 US expansion of the [^177^Lu]Lu-PSMA-617 indication after PSMAfore did not automatically make the wording of other regional authorizations identical. Similar distinctions apply to diagnostic agents, paediatric extrapolation, local preparation, and off-label use. Tables should therefore include the jurisdiction and verification date for regulatory statements. Global labels such as “approved” or “clinically available” are insufficient unless the intended region is specified.

The evidence priorities that follow from this analysis are concrete. Molecular-design studies should use paired chemistry comparisons and multiple sampling times rather than isolated high-uptake examples. Early human studies should report administered mass, metabolite data, lesion and organ kinetics, and uncertainty in dose estimates. Diagnostic trials should test lesion-level accuracy, reproducibility, management impact, or patient outcomes for a stated indication. Therapeutic development should progress from reproducible manufacturing and dosimetry to controlled, platform-specific trials rather than infer benefit from imaging. Multicentre programmes should include process transfer, inter-site comparability, and harmonized quantitative reporting. Finally, response frameworks should be validated against outcomes before PET-derived changes are treated as surrogate endpoints.

This Review has limitations. It is a targeted narrative synthesis, not a registered systematic review, so it cannot claim exhaustive retrieval or quantitative estimates of effect. Purposeful platform selection and reliance on published English-language sources create selection and publication bias. The scope spans molecular design, radiochemistry, manufacturing, regulation, and clinical trials; evidence hierarchies differ across those domains, and direct comparisons are often unavailable. Product-level regulatory status was verified for the United States and European Union through 9 August 2026 because the pivotal products discussed have centralized, publicly accessible regulator pages in those jurisdictions. The China National Medical Products Administration framework was checked at a general level (12), but a product-by-product Chinese or worldwide regulatory census was outside scope; absence from this Review must not be interpreted as non-authorization in China or elsewhere. Several emerging platforms are represented by small first-in-human or retrospective studies, and negative or unsuccessful development programmes are less likely to be published. These limits are reflected in the bounded evidence language and source-specific tables.

The field will advance most reliably when development decisions are tied to the uncertainty they are intended to reduce. A new chelator should be judged by chemical identity, stability, and comparative pharmacokinetics; a new imaging agent by diagnostic or decision performance; a therapeutic construct by tumour and organ exposure, safety, and controlled clinical outcomes; and a manufacturing change by demonstrated comparability. This discipline is less visually dramatic than a pipeline filled with targets and arrows, but it produces a more useful map for researchers, clinicians, and regulators.

## 9 Conclusion

PET radiopharmaceuticals should be evaluated as integrated products; radionuclides are not interchangeable appendages to a targeting vector. Target accessibility and heterogeneity, vector kinetics, linker and chelator chemistry, administered mass, radionuclide properties, manufacturing controls, dosimetry, and the clinical selection rule jointly determine whether a construct is useful. PSMA- and somatostatin receptor-directed platforms show that imaging can support effective radionuclide therapy when product, population, interpretation, and trial evidence are aligned. FAP/FAPI, HER2, CXCR4, and other emerging targets show why human uptake or early feasibility cannot substitute for diagnostic accuracy, controlled therapeutic outcomes, or reproducible manufacturing.

The practical implication is an evidence-gated development strategy. Each study should address the largest unresolved translational risk with a comparator and endpoint matched to the claim. Figures and tables should preserve product identity, evidence level, jurisdiction, and important negative findings without collapsing them into a universal pathway. Patient-specific dosimetry and molecular response assessment are valuable measurement frameworks, but outcome benefit and surrogate validity require prospective testing. Under these boundaries, molecular innovation can support clinically relevant exposure, reproducible production, and more evidence-aligned clinical decisions without overstating maturity.
